# Removal of neonicotinoids present in secondary effluents by ferrate(VI)-based oxidation processes

**DOI:** 10.1007/s11356-024-33167-3

**Published:** 2024-04-08

**Authors:** Francisco J. Real, Juan L. Acero, Esther Matamoros

**Affiliations:** Departamento de Ingeniería Química y Química Física, Facultad de Ciencias, Instituto Universitario de Investigación del Agua, Cambio Climático y Sostenibilidad (IACYS), Universidad de Extremadura, Avda. de Elvas S/N, 06006 Badajoz, Spain

**Keywords:** Neonicotinoids, Ferrate, Kinetics, Advanced oxidation processes, Water treatment

## Abstract

The persistence in the environment and possible harmful effects of neonicotinoid insecticides have raised some concerns, which have led to the proposal of various measures for their remediation. The aim of this work was to study the elimination of five neonicotinoids (thiamethoxam (THM), imidacloprid (IMI), clothianidin (CLO), thiacloprid (THC), and acetamiprid (ACE)) using ferrate (Fe(VI)) as the oxidizing agent. Firstly, second-order rate constants for the reactions of neonicotinoids with Fe(VI) were determined at different pHs. The most reactive compound was THC, with a rate constant of 400 ± 43 M^−1^ s^−1^ at pH 8 (the optimum pH considering the predominance of the most reactive species (HFeO_4_^−^) and the decreasing self-decomposition of Fe(VI) with pH), followed by CLO (10.7 ± 1.7 M^−1^ s^−1^), THM (9.7 ± 0.7 M^−1^ s^−1^), and IMI (2.5 ± 0.6 M^−1^ s^−1^). ACE did not significantly react with Fe(VI). The oxidation of the selected pollutants in secondary effluents by Fe(VI) was rather slow, and only THC could be efficiently removed. The presence of natural organic matter (NOM) exerted a negative influence on the removal of the neonicotinoids of moderate reactivity with Fe(VI) (CLO, THM, and IMI). The additional presence of peroxymonosulfate (Fe(VI)/PMS system) slightly increased the removal of neonicotinoids due to the formation of hydroxyl and sulfate radicals. Finally, the application of the Fe(VI)/sulfite system considerably increased the oxidation rate of the selected pollutants, with enhanced formation of hydroxyl and, especially, sulfate radicals. Overall, these results suggest that the Fe(VI)/sulfite system has significant potential to address environmental and health concerns associated with neonicotinoids in water sources with low NOM content.

**Graphical abstract:**

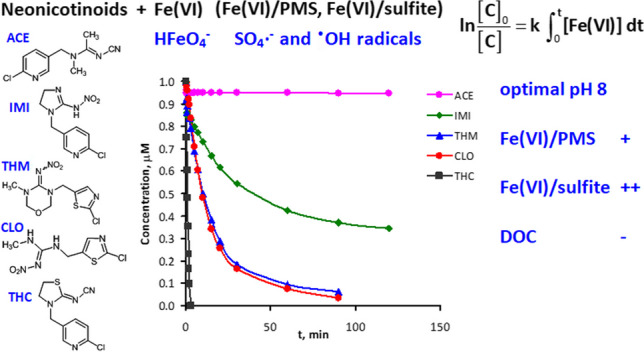

**Supplementary Information:**

The online version contains supplementary material available at 10.1007/s11356-024-33167-3.

## Introduction

Neonicotinoids, a new class of neuro-active insecticides, have been extensively used all over the world (Chen et al. 2019; Cho and Wei 2023). These insecticides present high efficacy and low cross-resistance and account for around 25% of the global pesticide market (Hladik et al. 2018). Due to the widespread use of neonicotinoids, many studies have reported their presence in water, soil, and food (Zhang et al. 2023a). Although their use has been restricted in many countries due to their potential harmful effects, these micropollutants were found in surface waters in regions of Europe and in many other aquatic systems throughout the world in concentrations in the order of ng/L to μg/L (Struger et al. 2017; Auteri et al. 2017). Moreover, neonicotinoids are compounds that have been considered of great concern by environmental agencies because of their potential risks to the aquatic environment, bees, and human health (Chen et al. 2019; Zhang et al. 2023a). For this reason, the monitoring of five of these neonicotinoids has appeared in several decisions of the European Union: thiamethoxam (THM), imidacloprid (IMI), clothianidin (CLO), thiacloprid (THC), and acetamiprid (ACE).

The elimination of neonicotinoids from water can be achieved by appropriate physical or chemical methods. Specifically, some advanced oxidation processes (AOPs) have demonstrated their efficiency in the elimination of these micropollutants, such as photocatalytic (Faisal et al. 2021; Wei et al. 2023), ozone-based (Real et al. 2022; Sales-Alba et al. 2023), or Fenton reaction–based (Boukhemkhem et al. 2023; Wei et al. 2023) processes. In this work, the degradation of these five neonicotinoids has been carried out by several oxidation systems involving ferrate (Fe(VI)), which is an environmentally friendly water treatment agent due to its high redox potential (2.2 V), excellent disinfectant properties, and capacity to improve coagulation efficiency (Sharma et al. 2016; Dong et al. 2018; Wang et al. 2021).

Fe(VI) exists in four different protonation states in aqueous solution (Eq. 1–3), and its reactivity varies significantly depending on its speciation (Rush et al. 1996; Sharma et al. 2001).

1 $${\text{H}}_{{3}} {\text{FeO}}_{{4}}^{ + } \, \rightleftarrows {\text{ H}}_{{2}} {\text{FeO}}_{{4}}^{{}} \, + {\text{ H}}^{ + } {\text{ pK}}_{{1}} { = 1}{\text{.5}}$$

2 $${\text{H}}_{{2}} {\text{FeO}}_{{4}}^{{}} \, \rightleftarrows {\text{ H}}_{{}} {\text{FeO}}_{{4}}^{ - } \, + {\text{ H}}^{ + } {\text{ pK}}_{{2}} { = 3}{\text{.5}}$$

3 $${\text{H}}_{{}} {\text{FeO}}_{{4}}^{ - } \, \rightleftarrows {\text{ FeO}}_{{4}}^{{2 - }} \, + {\text{ H}}^{ + } {\text{ pK}}_{{3}} { = 7}{\text{.2}}$$

In general terms, the oxidizing power of Fe(VI) increases and its stability decreases with decreasing pH due to a higher reactivity of the more protonated Fe(VI) species (Lee et al. 2008). However, HFeO_4_^−^ mainly exists rather than H_2_FeO_4_ with the highest oxidizing capacity under neutral or slightly basic environmental conditions (pH 6–10) (Kamachi et al. 2005), while FeO_4_^2−^ is known to be much less reactive than HFeO_4_^−^, and thus, its contribution is negligible (Lee et al. 2008). Therefore, it can be assumed that HFeO_4_^−^ is the main oxidizing species in systems involving Fe(VI).

In recent years, combinations of Fe(VI) with other chemicals such as hydrogen peroxide (H_2_O_2_), peroxymonosulfate (PMS, KHSO_5_ basis) and sulfite (SO_3_^2−^) have been proposed as innovative chemical oxidation technologies for the treatment of organic pollutants. Thus, Yu et al. (2023) established that the combination with PMS not only enhanced the stability of ferrate(VI) but also improved the removal rate of target pollutants. Also, Feng et al. (2017) demonstrated that a combination of PMS and Fe(VI) synergistically enhanced the removal of fluoroquinolones in comparison with Fe(VI) or PMS alone. Also, Wu et al. (2018) observed that the Fe(VI)/PMS system enhanced the degradation of atrazine compared to Fe(VI) or PMS alone, and its degradation efficiency was even higher than that of Fe(VI)/persulfate or Fe/H_2_O_2_ processes. In the Fe(VI)/PMS system, PMS promotes the reduction of Fe(VI) to Fe(III) and Fe(II) in solution. Then, Fe(II) and γ-Fe_2_O_3_ particles formed by coagulation of Fe(III) react with PMS to produce sulfate radical (SO_4_^−^), which subsequently can react with H_2_O to form hydroxyl radicals (^•^OH) (Wu et al. 2018). Therefore, SO_4_^−^ and ^•^OH are considered the main reactive oxygen species (ROS) in the Fe(VI)/PMS system. Similarly, Zhang et al. (2017) found that adding Na_2_SO_3_ to Fe(VI) could accelerate the degradation rate of organic pollutants. These organics could be completely removed within 30 s after adding Na_2_SO_3_, whereas the removal rate after adding Fe(VI) alone was less than 6%. Fe(VI) reacts with SO_3_^2−^ to generate sulfite radical (SO_3_^−^) and then SO_4_^−^ is produced through Eqs. (4)–(6) (He et al. 2022). Therefore, the oxidation capacity enhancement in the Fe(VI)/sulfite system may be attributed to the generation of more active species, such as SO_4_^−^ and ^•^OH. The optimum ratio of Fe(VI) to SO_3_^2−^ was determined to be around 1:4, since with greater excess of SO_3_^2−^, Fe(VI) reacts with SO_3_^−^ and reduces the formation of SO_4_^−^ (Sharma and Cabelli 2009).

4 $${\text{Fe(VI)}}\;{ + }\;{\text{SO}}_{3}^{2 - } \, \to {\text{ Fe(V) }} + {\text{ SO}}_{3}^{\cdot - }$$

5 $${\text{SO}}_{3}^{\cdot - } \;{ + }\;{\text{O}}_{2} \, \to {\text{ SO}}_{5}^{\cdot - }$$

6 $${\text{SO}}_{5}^{\cdot - } \; + \;{\text{SO}}_{3}^{2 - } \; \to {\text{ SO}}_{4}^{\cdot - } \; + \;{\text{SO}}_{4}^{2 - }$$

There is very little information in the literature about the oxidation of neonicotinoids pesticides by Fe(VI)-based systems. Only a few works focused on the oxidation of IMI have been found. Zhang and Jiang (2021) studied the removal by Fe(VI) of IMI among other emerging micropollutants, achieving 22–85% removal of IMI. Nitro and amino groups attached to the imidazole ring of IMI can be removed by Fe(VI) because of their strong ability for electrophilic substitution. Wang et al. (2022) also studied the removal of IMI by Fe(VI) and observed decreasing apparent rate constants from pH 6 to 9 (from 120 to 8.3 M^−1^ s^−1^). They monitored the main degradation intermediates and suggested a reaction pathway through the loss of the nitro group.

Due to the lack of information concerning the removal of neonicotinoids by ferrate-based oxidation processes, the present work was designed to investigate the degradation of the five neonicotinoids (THM, IMI, CLO, THC, and ACE) listed in the EU Decision 2018/840 by means of Fe(VI) and Fe(VI)-based AOPs, with the goal of efficiently removing these micropollutants from real water systems. The second-order rate constants of their reactions with Fe(VI) were determined. Furthermore, the influence of pH and Fe(VI) concentration on the degradation efficiency of the neonicotinoids was analyzed. In addition, the effect of the presence of organic and inorganic matter in secondary effluents on the efficiency of the process was evaluated. Finally, the improvement in the degradation of neonicotinoids produced by the Fe(VI)/PMS and Fe(VI)/sulfite systems was explored with the aim of evaluating the effect of the reactive oxygen species generated in these AOPs, as well as to find the most efficient advanced process to practically carry out the elimination of these compounds in real waters.

## Materials and methods

### Chemicals, reagents, and secondary effluents

Neonicotinoids thiamethoxam (THM, C_8_H_10_ClN_5_O_3_S), imidacloprid (IMI, C_9_H_10_ClN_5_O_2_), clothianidin (CLO, C_6_H_8_ClN_5_O_2_S), thiacloprid (THC, C_10_H_9_ClN_4_S), and acetamiprid (ACE, C_10_H_11_ClN_4_) were purchased from Sigma-Aldrich (Germany) at the highest purity available (99%). Table S1 of the “Supplementary information (SI)” lists the main physico-chemical parameters of the five neonicotinoids, as well as their chemical formulas and chemical structures. Fe(NO_3_)_3_·9H_2_O (99.9%) and PMS (≥ 47%, KHSO_5_ basis) were also purchased from Sigma-Aldrich. Na_2_SO_3_, tert-butanol (TBA), methanol (MeOH), humic acids (HAs), and other general reagents used in this research were provided by Panreac (Spain) of the highest available purity. Ultra-pure (UP) water with a specific resistance of 18.2 MΩ·cm was produced from a Milli-Q Water System (Millipore Iberica, Spain). Potassium ferrate (K_2_FeO_4_) was synthesized by the wet method (Li et al. 2005), through the oxidation of Fe(III) salts under strong alkaline conditions. Stock solutions of Fe(VI) (1–5 mM) were freshly prepared by dissolving a desired amount of potassium ferrate into a 5 mM K_2_HPO_4_/1 mM Na_2_B_4_O_7_·10H_2_O buffer solution (pH ≈ 9.1) and standardized spectrophotometrically at 510 nm (Ɛ = 1150 M^−1^ cm^−1^) (Sharma et al. 2001).

In addition to the experiments with UP water, two secondary effluents, SEA and SEB, collected in WWTPs located in Extremadura (southwestern Spain), were also used in different experiments. The secondary effluents were filtered through 0.45-µm filters and stored at 4 °C. The main quality parameters of SEA and SEB are summarized in Table S2.

### Experimental setup

Kinetics experiments of individual neonicotinoids were conducted with Fe(VI) in excess ([Fe(VI)]_0_: 50–100 μM) to determine the second-order rate constant of the reaction between Fe(VI) and each neonicotinoid. In a typical experiment, a 200-mL volume of buffered neonicotinoid solution (1.0 μM) was prepared in a batch reactor of 250 mL, which was located in a thermostatic bath at 20 °C. The pH was varied between 6.0 and 9.0 with 10 mM phosphate buffer. Each run was initiated by injecting into the flask the corresponding volume of a recently analyzed K_2_FeO_4_ stock solution to achieve the desired initial Fe(VI) concentration. The reaction mixtures were homogenized using a magnetic stirrer. At regular times, 1.5 mL of sample was rapidly transferred with a syringe into an HPLC vial containing 10 μL of thiosulfate (0.1 M) to stop the reaction and analyze the neonicotinoid concentration. At the same time, 3 mL of the solution was sampled to determine the residual Fe(VI) concentration by the ABTS method.

Similar procedures were conducted in the following stages, but initially, 1 µM of each of the five neonicotinoids were dissolved together in different water systems (UP water, SEA, SEB and HA water solutions). UP water experiments were performed at different pHs (6.0–9.0) and by varying the initial concentration of Fe(VI) (25–100 μM). While HA solutions (2–10 mg/L) with contaminants were buffered at pH 8, experiments performed with SEA and SEB were not buffered (pH around 8). When using the Fe(VI)/PMS or Fe(VI)/sulfite systems, the corresponding reagent, PMS or Na_2_SO_3_, was also added to the reactor to reach the desired initial concentration (from 200 to 400 μM). Quenching experiments were carried out to determine the contribution of reactive species, using TBA and MeOH as radical scavengers. Similarly to kinetics experiments, two samples were withdrawn from the reactor at selected times to analyze neonicotinoids and Fe(VI) concentration.

The concentration of each neonicotinoid was determined by HPLC, following the analytical methods described in previous works (Acero et al. 2019; Real et al. 2022). The details are described in Text S1. The concentration of Fe(VI) was determined by the ABTS method (Lee et al. 2005). The characterization of the real water matrices was carried out following the Standard Methods (Clesceri et al. 1989). Dissolved organic carbon (DOC) was determined using a TOC-multi N/C 3100 analyzer (Analytik Jena, Germany).

## Results and discussion

### Kinetics of the oxidation of the neonicotinoids with ferrate(VI)

Experiments of degradation by Fe(VI) of five neonicotinoids (THM, IMI, CLO, THC, and ACE) individually dissolved in UP water have been carried out at 20 °C with the aim of determining their second-order rate constant values. The solutions were buffered at different pHs, ranging between 6 and 9. The initial concentration of each neonicotinoid was 1 μM and the initial concentration of Fe(VI) ranged from 50 to 100 μM. Table 1 compiles the conditions applied for each experiment, as well as the neonicotinoid removal percentages achieved and the Fe(VI) decay after 15 min of reaction.

**Table 1:** Degradation of neonicotinoids by Fe(VI) in UP water: removal percentages and Fe(VI) decay obtained after 15 min of reaction

pH	[Fe(VI)]_0_, μM	X_THM_, %	X_IMI_, %	X_CLO_, %	X_THC_, %	X_ACE_, %	Fe(VI) decay, %
6	50	4.3	3.2	12.8	73.5	-	91.8
6	100	27.6	16.2	28.0	100	< 1	96.3
6.5	50	9.0	3.7	13.9	> 99	-	88.3
7	50	15.7	3.8	26.2	100	-	79.8
7	100	38.2	27.6	49.0	100	5.4	90.3
7.5	50	15.9	6.1	24.4	100	-	66.2
8	50	24.0	10.4	28.2	100	-	31.2
8	100	61.7	33.2	65.7	100	5.1	40.2
9	50	7.2	1.7	8.1	56.6	-	6.1
9	100	13.2	6.4	15.7	100	< 1	10.9

Initial concentration of each neonicotinoid = 1 μM; T = 20 °C

**Fig. 1:**
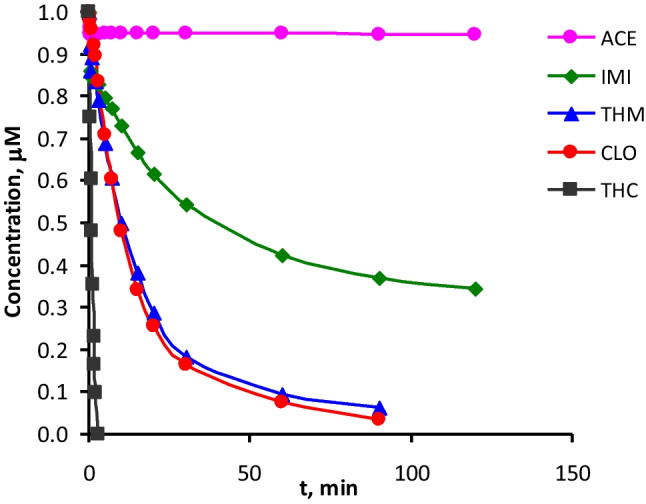
Evolution of the degradation of the five neonicotinoids by Fe(VI) in UP water. Experimental conditions: Initial concentration of each neonicotinoid = 1 μM; T = 20 °C; pH = 8; [Fe(VI)]_0_ = 100 μM except for THC (50 μM)

According to the results exposed in Table 1, THC was significantly the most reactive compound, being eliminated after 15 min of reaction at pH between 6.5 and 8 with 50 μM of Fe(VI), while ACE did not react with Fe(VI) under any condition. The reactivity trend observed for the five neonicotinoids was THC >  > CLO ≥ THM > IMI >  > ACE. The same trend can be clearly observed in Fig. 1, which depicts the neonicotinoids decay for experiments carried out at pH 8 and with an initial Fe(VI) concentration of 100 μM (except for THC, conducted with [Fe(VI)]_0_ = 50 μM, since, when using 100 μM of Fe(VI), THC was not detected after 15 s of reaction). It can be seen that both CLO and THM can also be effectively eliminated at pH 8 after 90 min of reaction, while IMI was only partially removed.

Regarding the influence of pH, two conclusions can be drawn from the data collected in Table 1: it can be observed that the optimum pH for the degradation of the neonicotinoids was between 7 and 8; likewise, Fe(VI) decay was favored at low pH. The influence of this parameter on the efficiency of the process will be discussed further in the next section.

In order to evaluate the actual reactivity of Fe(VI) with each neonicotinoid at different pHs, the residual concentrations of both the organic compound (C) and Fe(VI) were determined with reaction time.

Assuming second-order kinetics for the reaction of neonicotinoids with Fe(VI) (reaction (7)), as previously found for the oxidation of different classes of micropollutants (Liu et al. 2019; Wang et al. 2022), Eq. (8) allows determining the second-order rate constants, *k*:

7 $${\text{Neonicotinoid + Fe(VI) }}\mathop{\longrightarrow}\limits^{{\text{k}}}{\text{ Products}}$$

8 $$\ln \frac{{\left[ C \right]_{0} }}{{\left[ C \right]_{{}} }} = k_{{}} \int_{0}^{t} {[Fe(VI)]} dt$$

The integral term was evaluated for each reaction time from the experimental values of Fe(VI) concentration versus time. The values of *k* obtained for each experiment by linear regression of the terms of Eq. (8) are collected in Table 2. As can be seen, the rate constant values for all the micropollutants presented similar values in the pH range 6–8, but decreased strongly at pH 9, which confirms that the optimum pH of Fe(VI) oxidation is neutral or slightly basic and that the reactivity of HFeO_4_^−^ is higher than that of FeO_4_^2−^. Moreover, the trend of reactivity of the neonicotinoids commented above is also confirmed (THC >  > CLO ≥ THM > IMI). The almost 0 decrease of ACE concentration in the experiments carried out prevented the determination of any rate constant for ACE, suggesting a *k* value lower than 1 M^−1^ s^−1^. The rate constant values at 20 °C proposed for IMI (in the range 2.4–2.8 M^−1^ s^−1^ for pH 6–8) are lower than those obtained at 25 °C by Wang et al. (2022) (e.g., 24 ± 8 M^−1^ s^−1^ at pH 8). Apart from the higher temperature, an explanation for this difference in the rate constants’ values might be that Wang et al. (2022) considered only the first seconds of the reaction when the consumption of Fe(VI) was very low. According to these results, THC can be rapidly eliminated by Fe(VI) as an oxidizing agent, while CLO and THM can also be removed at optimum conditions.

**Table 2:** Second-order rate constants k (M^-1^ s^-1^) for the reaction of Fe(VI) with THM, IMI, CLO and THC at different pH

pH	[Fe(VI)]_0_, μM	THM		IMI		CLO		THC	
6	50	7.5	8.1 ± 0.6	2.8	2.6 ± 0.2	13.0	14.0 ± 1.1	171	171 ± 4
	100	8.6		2.4		15.1		-	
6.5	50	7.1	7.1 ± 0.4	2.8	2.8 ± 0.7	16.3	16.3 ± 0.3	243	243 ± 6
7	50	12.5	13.7 ± 1.2	2.5	2.7 ± 0.2	18.5	18.7 ± 0.2	507	507 ± 21
	100	14.9		3.0		19.0		-	
7.5	50	6.9	6.9 ± 0.3	2.4	2.4 ± 0.1	11.7	11.7 ± 0.2	572	572 ± 26
8	50	9.0	9.7 ± 0.7	2.0	2.5 ± 0.6	9.0	10.7 ± 1.7	400	400 ± 43
	100	10.4		3.1		12.4		-	
9	50	2.2	2.3 ± 0.1	0.7	0.7 ± 0.1	2.6	3.2 ± 0.6	12.6	22.6 ± 0.6
	100	2.4		0.8		3.8		-	

The reactivity of neonicotinoids with Fe(VI) can be explained by their chemical structure (Table S1). Generally, Fe(VI) reacts with electron-rich moieties (ERM) in micropollutants (e.g., phenol and aniline), organosulfur compounds, and deprotonated amines (Zhang et al. 2020). Thus, Fe(VI) reacts predominantly with sulfur-containing neonicotinoids (THC, CLO, and THM with a thiazole ring), and the reactivity with the remaining neonicotinoids is rather low (IMI) or inappreciable (ACE). The absence of the electron-withdrawing Cl moiety in the thiazole ring of THC promotes its reactivity compared with CLO and THM. In addition, Fe(VI) could attack to the N-nitro moiety of CLO, THM, and IMI, causing the substitution of the –NO_2_ group by hydrogen. Previous investigations have proposed that the disproportionate N–N bond of nitro and amino groups attached to the imidazole ring of IMI can be weakened by an electron transfer oxidation reaction with Fe(VI), leading to the release of NO_3_^−^ (Zhang and Jiang 2021; Wang et al. 2022). However, the low reactivity of IMI indicates that the N-nitro group is less reactive than the thiazole ring. Finally, pyridine rings with deactivating Cl atoms and N-cyano imine moieties present very low reactivity with Fe(VI).

### Degradation of a mixture of neonicotinoids by Fe(VI) in UP water: effect of the pH

**Fig. 2:**
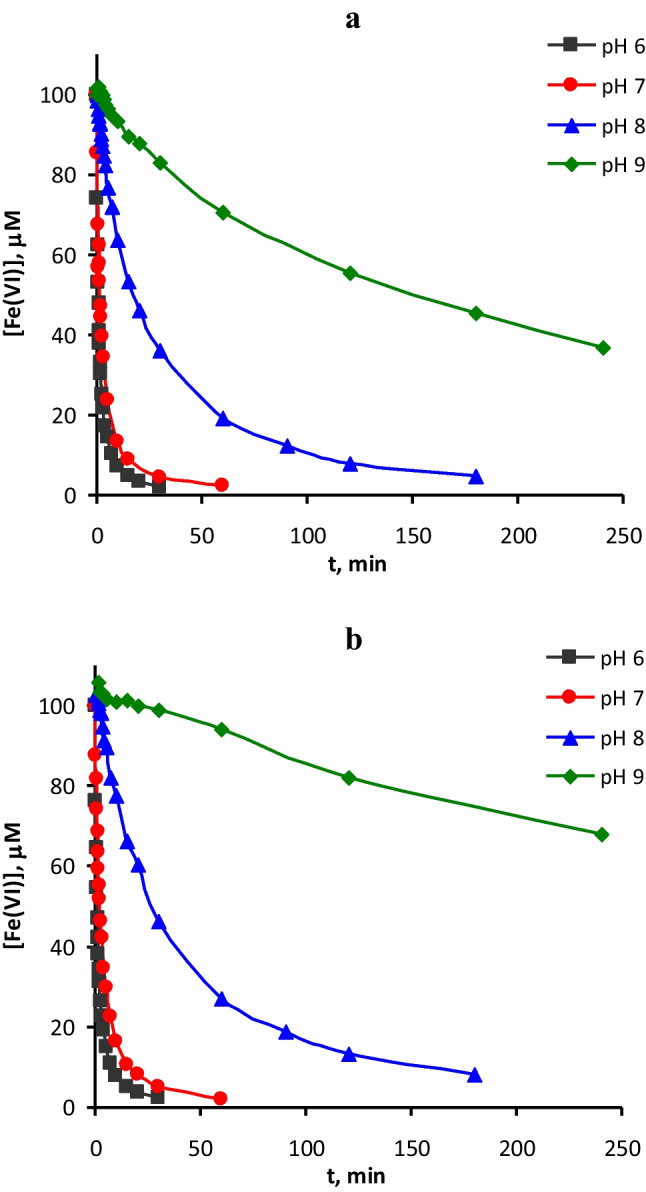
Decay of Fe(VI) with reaction time in experiments performed in UP water at different pHs **a** with a mixture of five neonicotinoids and **b** without contaminants. Experimental conditions: Initial concentration of each neonicotinoid = 1 μM; [Fe(VI)]_0_ = 100 μM; T = 20 °C

A series of four degradation experiments by Fe(VI) was carried out on a mixture of the five neonicotinoids dissolved together in UP water (initial concentration of each micropollutant of 1 μM) at pH between 6 and 9. The initial Fe(VI) concentration applied was 100 μM. The decay of Fe(VI) was also followed throughout reaction time and the results are depicted in Fig. 2a. The effect of pH on the decomposition of Fe(VI) is very noticeable, being faster at lower pH due to its self-decomposition occurring at neutral and acidic pH (Lee et al. 2008). Figure 2b shows the self-decay of Fe(VI) in a set of blank experiments with an initial concentration of 100 μM, without adding any micropollutant. In addition, Figure S1 of the SI shows the decay of Fe(VI) throughout the experiments of degradation of THM listed in Table 1 with [Fe(VI)]_0_ = 50 μM at each pH assayed. Very similar results were obtained in these previous experiments of degradation of each individual neonicotinoid at different pHs and also when no compound was added, indicating that self-decomposition is the main cause of the decrease in the concentration of Fe(VI), and that the presence of micropollutants in low concentration hardly affects its self-decomposition (Liu et al. 2019). Therefore, the fast self-decomposition of HFeO_4_^−^, the main Fe(VI) species in the pH range 6–7, is responsible for the small amount of Fe(VI) available for target compound oxidation at acidic pHs. However, at higher pH, the self-decay is slower due to the predominance of the deprotonated and less reactive species FeO_4_^2−^, thus increasing the availability of Fe(VI) for micropollutant oxidation.

**Fig. 3:**
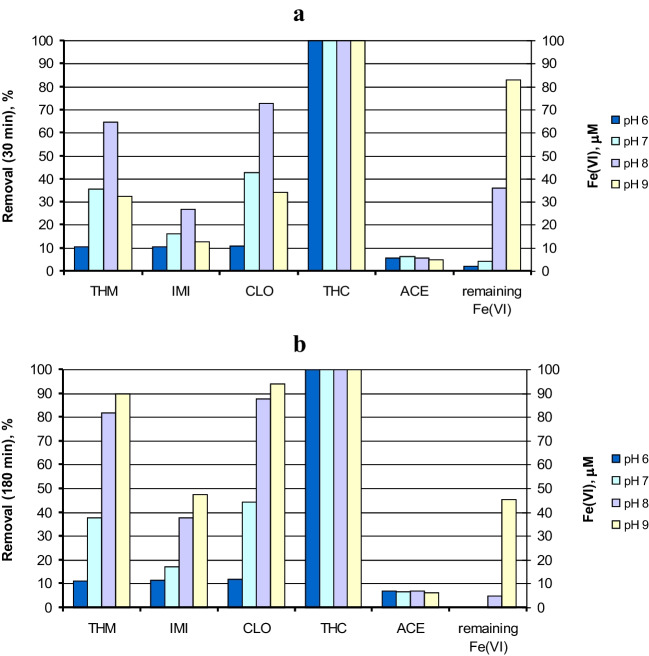
Degradation of a mixture of neonicotinoids by Fe(VI) in UP water at different pHs: removal percentages obtained (%) and residual concentration of Fe(VI) after **a** 30 min and **b** 180 min of reaction. Experimental conditions: Initial concentration of each neonicotinoid = 1 μM; [Fe(VI)]_0_ = 100 μM; T = 20 °C

The self-decomposition rate of Fe(VI) plays a very important role in the degradation of the micropollutants. Figure 3 shows the influence of pH on micropollutant degradation. Figure 3a shows the removals reached after 30 min of reaction for each neonicotinoid, as well as the residual concentration of Fe(VI) at this time (already depicted in Fig. 2a), while Fig. 3b shows the same parameters taken at 180 min of reaction. In addition, Fig. S2 shows the evolution of the five neonicotinoids with reaction time during Fe(VI) oxidation in UP water at different pHs. According to Fig. 3a, pH 8 seems to be the optimum for the degradation of the neonicotinoids with intermediate reactivity (CLO, THM, and IMI). Almost no residual Fe(VI) was detected after 30 min at pH 6 and 7; therefore, the reaction practically stopped from this time on. This fact can be confirmed in Fig. 3b, in which almost no improvement in the removal of the compounds occurred for 180 min of reaction time in experiments conducted at pH 6 and 7. In addition, the 180-min removals reached at pH 9 were higher than those at pH 8. However, according to the results exposed in Figure S2, it takes over 120 min of reaction to equalize the removals at pH 8 and 9. The degradation rate for all the compounds was clearly faster at pH 8 during the first hour of the reaction. The slower degradation rate at pH 9 agrees with the low rate constant values obtained at high pH. A similar influence of pH was observed during Fe(VI) oxidation of other micropollutants such as parathion (Liu et al. 2019) or sulfachloropyridazine (Sun et al. 2019).

In conclusion, the optimum pH for the degradation of neonicotinoids is pH 8, since at pH 9 the reactivity of Fe(VI) as an oxidizing agent of micropollutants decreases significantly (Lee et al. 2008) and at pH ≤ 7 the self-decay rate of Fe(VI) is fast and its oxidant exposure diminishes (Sun et al. 2019). Therefore, at pH 8 an optimum compromise between the reactivity of the Fe(VI) species and their self-decomposition is reached.

### Degradation of a mixture of neonicotinoids by Fe(VI) in secondary effluents

**Fig. 4:**
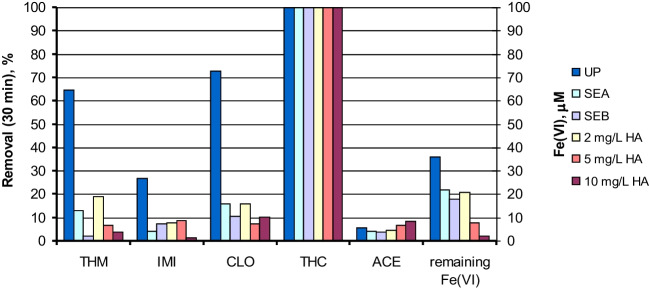
Degradation of a mixture of neonicotinoids by Fe(VI) in UP water, secondary effluents and in the presence of HA: removal percentages obtained (%) and residual concentration of Fe(VI) (μM) after 30 min of reaction. Experimental conditions: Initial concentration of each neonicotinoid = 1 μM; pH ≈ 8; [Fe(VI)]_0_ = 100 μM; T = 20 °C

In order to determine the role of the natural organic matter (NOM) present in the water, a new series of oxidation experiments by Fe(VI) was carried out with the five neonicotinoids simultaneously dissolved (1 μM of each one) in different water matrices: UP water, two secondary effluents (SEA and SEB), and water solutions of 2, 5, and 10 mg/L of HA, selected as a representative of the NOM present in real water samples. All the experiments were conducted at pH 8, or at the natural pH of the secondary effluent (also at pH around 8). Figure 4 shows the removal percentages obtained for each neonicotinoid after 30 min of reaction, as well as the residual concentration of Fe(VI) in every water system tested. The presence of NOM exerted a negative influence on the removal of the neonicotinoids of moderate reactivity with Fe(VI) (CLO, THM, and IMI), whose removal percentages were at their maximum value in UP water and were reduced by increasing the NOM content of the water matrix. However, THC was completely removed in all the experiments, although the time required was different in each water system used. Figure S3 shows the evolution of THC in these experiments, being the time required for complete THC removal in the secondary effluent SEB (DOC = 9.4 mg/L) 5 min, higher than that in the experiment conducted in UP water (1 min) or in SEA (3 min, DOC = 4.3 mg/L). Similarly, it took around 10 min to completely remove THC in UP water with 10 mg/L of HA. Finally, ACE removal percentages hovered around an insufficient 4–9%, with no signs of the influence of the NOM content. TOC was also measured along the runs as a NOM content indicator, but its final removal percentages were always below 10%. It is also remarkable that the residual concentration of Fe(VI) after 30 min (Fig. 4) is in accordance with the NOM present in the water system. In this case, NOM competes with neonicotinoids for the existing Fe(VI), and promotes a fast Fe(VI) reduction (Horst et al. 2013). Consequently, the presence of NOM in the water inhibits the degradation of the neonicotinoids, among which only THC continues to be efficiently removed. In addition, the presence of bicarbonate ions (alkalinity in Table S2) might exert some inhibitory effect on the degradation of neonicotinoids (Liu et al. 2019). Fe(VI) can be a more efficient oxidant than non-selective ^•^OH for degrading some neonicotinoids such as THC. Thus, in other advanced oxidation processes, such as UV-based or ozone-based technologies (Acero et al. 2019; Real et al. 2022), THC was one of the most refractory neonicotinoids. Therefore, Fe(VI) is postulated as a promising technology for the removal of THC, even in secondary effluents with high NOM content.

### Degradation of a mixture of neonicotinoids by ferrate-based oxidation processes

Next, the Fe(VI)-based AOPs Fe(VI)/PMS and Fe(VI)/sulfite were applied to a mixture of the five neonicotinoids. Preliminary experiments were performed with PMS or sulfite alone. As can be observed in Fig. S4, the degradation of neonicotinoids with single PMS was typically below 10% after 30 min of reaction, even when a high concentration of PMS (400 µM) was used. Only THC could be partially degraded by PMS, reaching 15 and 27% removal for PMS concentrations of 200 and 400 µM, respectively. The presence of sulfite did not affect the concentration of neonicotinoids.

**Fig. 5:**
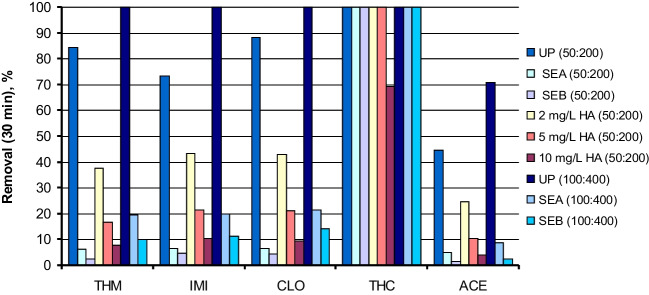
Degradation of a mixture of neonicotinoids by the Fe(VI)/PMS system in UP water, secondary effluents and in the presence of HA: removal percentages of each neonicotinoid (%) after 30 min of reaction. Experimental conditions: Initial concentration of each neonicotinoid = 1 μM; pH ≈ 8; T = 20 °C; two initial Fe(VI):PMS concentration settings used: 50:200 μM and 100:400 μM

To investigate the efficiency of the Fe(VI)/PMS process for the removal of neonicotinoids, different experiments were performed at pH around 8 by varying the dose of Fe(VI) and PMS and the water matrix used. The initial ratio of Fe(VI):PMS concentration was kept at 1:4 in all the experiments, since the excessive addition of PMS could decrease the degradation of micropollutants due to the reaction between PMS and ROS (i.e., SO_4_^−^ and ^•^OH) (He and Zhao 2023). The results obtained in the experiments performed in UP water are depicted in Fig. S4. In addition, Fig. 5 shows the removal percentages obtained for each neonicotinoid after 30 min of reaction. An important improvement can be observed in the degradation of the neonicotinoids by the Fe(VI)/PMS system in UP water compared to single Fe(VI) oxidation, suggesting a synergistic effect between Fe(VI) and PMS. The high removals achieved for these compounds are especially noteworthy when using initial Fe(VI) and PMS concentrations of 100 and 400 μM, respectively, reaching 100% removal after 30 min for four of the five neonicotinoids. Thus, by comparing the results from Fig. 4 (single Fe(VI)) and Fig. 5, the removal percentage of, i.e., IMI increased from 26.8 to 100% due to the additional presence of PMS and the subsequent generation of SO_4_^−^ and ^•^OH (Wu et al. 2018; He et al. 2022). ACE was the most refractory micropollutant and could be only partially removed due to its low reactivity with Fe(VI) and ^•^OH (Real et al. 2022).

To analyze the effect of pH on the Fe(VI)/PMS process, experiments were carried out in UP water at different pHs with an initial concentration of Fe(VI) and PMS of 50 and 200 μM, respectively. According to the results shown in Fig. S5, the optimum pH for the removal of selected neonicotinoids was pH 8, except for THC, which was more efficiently oxidized at pH 7. Self-decomposition of Fe(VI) is very fast at acidic pH, while at pH above 9.0, the predominant species is FeO_4_^2−^, which is very stable, and thus, the activation of PMS to generate ROS is hindered (Wu et al. 2018). Therefore, the Fe(VI)/PMS process is feasible for degrading neonicotinoids at neutral or slightly basic pH. Similarly, previous studies have found that the optimum pH range for the degradation of micropollutants by the Fe(VI)/PMS process is 5–8 (Wu et al. 2018; He et al. 2022; He and Zhao 2023).

The relative contribution of SO_4_^−^ and ^•^OH to neonicotinoid degradation was examined in quenching experiments performed in the presence of TBA or MeOH (Fig. S6). Since the reactivity of TBA with ^•^OH (k_•OH-TBA_ = 6 × 10^8^ M^−1^ s^−1^) (Buxton et al. 1988) is around three orders of magnitude higher than that with SO_4_^−^ (k_SO4·-TBA_ = 8 × 10^5^ M^−1^ s^−1^) (Neta et al. 1988), TBA reacts predominantly with ^•^OH. However, MeOH can scavenge both ^•^OH and SO_4_^−^ (rate constants for reactions with ^•^OH and SO_4_^−^ of 9.7 × 10^8^ and 2.5 × 10^7^ M^−1^ s^−1^, respectively) (Buxton et al. 1988; Neta et al. 1988). The degradation efficiency of neonicotinoids significantly decreased in the presence of radical scavengers, being the inhibition effect of MeOH greater than that of TBA. Hence, both SO_4_^−^ and ^•^OH are the predominant reactive species responsible for THM, IMI, CLO, and ACE degradation by the Fe(VI)/PMS process. THC was likely oxidized by Fe(VI) and radicals due to its high reactivity with HFeO_4_^−^. High-valent iron species can be partially scavenged by MeOH and could also contribute to micropollutant degradation. However, in excess of PMS (ratio Fe(VI):PMS of 1:4), the dominant reactive species are ^•^OH and SO_4_^−^ (Zhang et al. 2023b).

The presence of NOM in SEA and SEB exerted a very negative effect on the Fe(VI)/PMS process, reaching similar results to the experiments carried out with Fe(VI) alone after 30 min of reaction (Fig. 4 and Fig. 5). The effect of the presence of HA, being negative, led to a lower decrease in the removal of the neonicotinoids. Only THC, the most reactive neonicotinoid, was completely degraded with the selected oxidant doses in almost any water system. The presence of NOM at high concentrations typically hinders the oxidation of micropollutants by direct competition with ROS (He and Zhao 2023). Similarly, the presence of carbonate/bicarbonate ions in the secondary effluents might inhibit the degradation of neonicotinoids, since carbonate/bicarbonate react with ^•^OH and SO_4_^−^ to produce carbonate radicals with weaker oxidation capacity (Zhang et al. 2023b). Probably, a significant increase in Fe(VI) and PMS doses would lead to an increase in the removal efficiency of neonicotinoids dissolved in these secondary effluents, but this effect has not been investigated further.

**Fig. 6:**
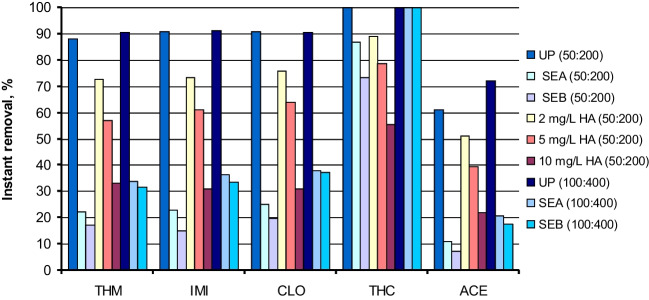
Degradation of a mixture of neonicotinoids by the Fe(VI)/sulfite system in UP water, secondary effluents and in the presence of HA: instant removal percentages of each neonicotinoid. Experimental conditions: Initial concentration of each neonicotinoid = 1 μM; T = 20 °C; pH ≈ 8; two initial Fe(VI):sulfite concentration settings used: 50:200 μM and 100:400 μM

The Fe(VI)/sulfite system is based on the reduction of Fe(VI) by sulfite, leading to intermediate reactive species, such as Fe(V) and SO_3_^−^, as well as the secondary radicals SO_4_^−^ and ^•^OH (Zhang et al. 2017; He et al. 2022). The generation of all these reactive species and the reaction with the micropollutants present are near-instantaneous, typically in the order of seconds (Yang et al. 2022). Figure 6 shows the instant removal percentages of the five neonicotinoids achieved in the experiments carried out with the Fe(VI)/sulfite system in different water matrices. The initial concentration ratio of Fe(VI):sulfite was kept at 1:4 in all the experiments, which was found to be the optimum ratio (Sharma and Cabelli 2009; Sun et al. 2018). The initial dosage of reagents applied in UP water exerted a limited influence on the removal percentage of THM, IMI, CLO (around 90%), and THC (completely degraded). However, the influence of the Fe(VI) and sulfite dosage (at a ratio of 1:4) was positive in the case of the less reactive neonicotinoid ACE, reaching a promising 60–72% removal in UP water. Gao et al. (2020) also found that methyl phenyl sulfoxide degradation by Fe(VI) alone was negligible, but the presence of sulfite considerably accelerated its removal up to a 90% within 30 s, with the involvement of sulfate radicals.

To examine the effect of pH (6–9) on the efficiency of the Fe(VI)/sulfite process, experiments were conducted in UP water at a fixed Fe(VI) and sulfite dosage of 50 and 200 μM, respectively. As can be observed in Fig. S7, the optimum pH for the removal of selected neonicotinoids was pH 8. These results could be attributed to the relatively high stability of Fe(VI) at pH 8–9 and to the fact that the reaction of Fe(VI) with sulfite became slow at higher pH (Sharma 2010). Similarly, Sun et al. (2018) found that the degradation of DEET by the Fe(VI)/sulfite system was faster at pH 8. Therefore, the Fe(VI)/sulfite process provides an alternative for removing neonicotinoids under slightly alkaline conditions.

According to the results obtained in the experiments performed in the presence of radical scavengers (Fig. S8), the degradation efficiency of the Fe(VI)/sulfite system slightly decreased in the presence of TBA. However, the inhibition effect of MeOH was much stronger. Since 5 mM TBA can almost completely scavenge ^•^OH, but hardly inhibit SO_4_^−^ (Sun et al. 2018), ^•^OH might play a minor role in the degradation of neonicotinoids by the Fe(VI)/sulfite system, being SO_4_ − the main reactive radical species. According to a previous study, although Fe(IV)/Fe(V) and SO_4_^−^/^•^OH were responsible for iopamidol degradation, SO_4_^−^ was identified as the main oxidant at the molar ratio of [sulfite]_0_/[Fe(VI)]_0_ ≥ 1.0 (Yang et al. 2022). Similarly, Zhao et al. (2023) found that sulfite accelerated the degradation of PAHs by undergoing a swift reaction with Fe(VI), leading to the formation of Fe(V) and SO_4_^−^. It can be observed in Fig. S8 that the degradation efficiency of neonicotinoids significantly decreased in deoxygenated conditions after purging with nitrogen. These results confirm that dissolved oxygen plays an important role in the formation of SO_4_^−^ in the Fe(VI)/sulfite system according to reactions (4–6).

In the case of experiments carried out in secondary effluents (Fig. 6), the effect of the NOM content was negative, leading to the following efficiency trend, UP water > HA solutions > SEA > SEB, although the removal percentages achieved were higher than those of Fe(VI) and Fe(VI)/PMS with similar dosages, and can be considered as moderate. Thus, the instantaneous removal percentages of THM, IMI, and CLO were around 30–38% when applying Fe(VI) and sulfite dosage of 100 and 400 μM, respectively, quite higher than the removal percentages at 30 min obtained for the Fe(VI)/PMS system, in the range of 10–22%. The inhibition observed in secondary effluents can be explained by the competition of NOM and some ions (such as chloride and bicarbonate) present in real water with neonicotinoids for SO_4_^−^.

## Conclusions

Fe(VI) was an excellent option for the degradation of THC. On the contrary, the oxidation of ACE by Fe(VI) was practically negligible at any pH. The order of reactivity found for the degradation by Fe(VI) of the five neonicotinoids tested was THC > CLO > THM > IMI > ACE. The best results were obtained at pH 8, which is a compromise value between the extent of self-decomposition of Fe(VI) and the predominance of the most reactive species, HFeO_4_^−^. The presence of NOM exerted a negative effect on pollutant removal, being THC the only neonicotinoid efficiently removed by Fe(VI) in secondary effluents.

The implementation of the Fe(VI)/PMS and Fe(VI)/sulfite systems caused a significant increase in the degradation of selected neonicotinoids through the generation of ROS, such as SO_4_^−^ and ^•^OH. The optimum pH for the removal of selected neonicotinoids by Fe(VI)/PMS and Fe(VI)/sulfite was pH 8. Specifically, the additional presence of sulfite accelerated the degradation rate of neonicotinoids through instant reactions, reaching the highest removal levels among the conditions tested. In the Fe(VI)/sulfite process, SO_4_^−^ was the main reactive radical species. These results suggest that the Fe(VI)/sulfite system may present a viable and environmentally friendly strategy to efficiently remove neonicotinoids from contaminated water. Due to the negative effect of NOM on the efficiency of these Fe(VI)-based AOPs, it is recommended to increase the dosage of reagents in the treatment of secondary effluents with non-negligible NOM content.

## Supplementary Information

Below is the link to the electronic supplementary material.

Supplementary file1 (DOC 221 KB)

## Data Availability

All data generated or analyzed during this study are included in this published article.
