# Supplementary material for: Removal of neonicotinoids present in secondary effluents by ferrate(VI)-based oxidation processes
Source: Environ Sci Pollut Res Int. 2024 Apr 8;31(20):29684–94. doi: 10.1007/s11356-024-33167-3 (PMC11512836; doi:10.1007/s11356-024-33167-3)
Supplement: Supplementary file 1 — Supplementary file1 (DOC 221 KB) [file 11356_2024_33167_MOESM1_ESM.doc]

Supporting information for

**Elimination of neonicotinoids present in secondary effluents by ferrate(VI)-based advanced oxidation processes**

Francisco J. Real*, Juan L. Acero and Esther Matamoros

Departamento de Ingeniería Química y Química Física, Instituto Universitario de Investigación del Agua, Cambio Climático y Sostenibilidad (IACYS), Facultad de Ciencias, Universidad de Extremadura, Avda. de Elvas s/n, 06006-Badajoz, Spain.

*Author to whom correspondence should be addressed. e-mail: fjreal@unex.es Fax number: +34 924289385. Tel. number: +34 924289384

Table of contents

Text S1 Chromatographic methods for the analysis of micropollutants p. S3

Table S1 Physico-chemical properties of selected neonicotinoids p. S4

Table S2 Quality parameters of selected secondary effluents p. S5

Figure S1 Decay of Fe(VI) with reaction time in experiments of oxidation of THM by Fe(VI) in UP water at different pH p. S6

Figure S2 Evolution of (A) THM, (B) IMI, (C) CLO, (D) THC and (E) ACE with reaction time during Fe(VI) treatment in UP water at different pH p. S7

Figure S3 Evolution of THC with reaction time during Fe(VI) treatment in UP water, secondary effluents and in the presence of HA p. S10

Figure S4 Evolution of (A) THM, (B) IMI, (C) CLO, (D) THC and (E) ACE with reaction time during Fe(VI)/PMS treatment in UP water with different concentrations of reagents p. S11

Figure S5 Evolution of (A) THM, (B) IMI, (C) CLO, (D) THC and (E) ACE with reaction time during Fe(VI)/PMS treatment in UP water at different pH p. S14

Figure S6 Evolution of (A) THM, (B) IMI, (C) CLO, (D) THC and (E) ACE with reaction time during Fe(VI)/PMS treatment in UP water in the presence of the radical scavengers TBA and MeOH p. S17

Figure S7 Degradation of neonicotinoids by the Fe(VI)/sulfite system in UP water at different pH: instant removal percentages of each neonicotinoid p. S20

Figure S8 Degradation of neonicotinoids by the Fe(VI)/sulfite system in UP water in the presence of the radical scavengers TBA and MeOH and under N_2_ atmosphere: instant removal percentages of each neonicotinoid p. S21

**Text S1.** Chromatographic methods for analysis of the micropollutants

The concentration of each neonicotinoid was determined by HPLC, using a Waters Chromatograph (Alliance 2695) equipped with a 2998 Photodiode Array Detector and a Phenomenex Kinetex® C18 Column (5μm, 150mm×4.6mm). The analytical method was adapted from previous works (Acero et al. 2019; Real et al. 2022). The mobile phase consisted of a mixture of methanol and 10 mM o-phosphoric acid aqueous solution, with a flow rate of 1 mL min^-1^ and the injection volume was 100 μL. While the analysis of individual neonicotinoids was performed in isocratic mode, the mixtures of the five neonicotinoids were analyzed in gradient mode. The methanol percentage in the isocratic mobile phase was 25% for THM, 30% for IMI, 40% for ACE and CLO, and 45% for THC. The selected detection wavelengths were 253, 271, 248, 267 and 244 nm for THM, IMI, ACE, CLO and THC, respectively. Methanol (A) and 10 mM o-phosphoric acid aqueous solution (B) percentages in gradient mode were the following:

Time (min) A (%) B (%)

0 25 75

3.5 25 75

8.5 45 55

12 45 55

14 25 75

18 25 75

The selected detection wavelengths in gradient mode were 253 nm for TMX, 280 nm for ICP, 267 nm for CTD, and 236 nm for AMP and TCP. The limit of quantification of the neonicotinoids was 0.02 μM and the limit of detection was 0.006 μM.

**Table S1** Physico-chemical properties of selected neonicotinoids

| **Neonicotinoid** | **Chemical Structure** | **MW, g/mol** | **pK_a_** | **log K_ow_** | **V_m_, cm^3^** |
| --- | --- | --- | --- | --- | --- |
| Thiamethoxam  (THM)  C_8_H_10_ClN_5_O_3_S |  | 291.71 | - | -1.16 | 170.2 |
| Imidacloprid  (IMI)  C_9_H_10_ClN_5_O_2_ |  | 255.67 | 11.1 | -0.43 | 160.1 |
| Clothianidin  (CLO)  C_6_H_8_ClN_5_O_2_S |  | 249.68 | - | 0.40 | 147.8 |
| Thiacloprid  (THC)  C_10_H_9_ClN_4_S |  | 252.73 | - | 0.55 | 177.4 |
| Acetamiprid  (ACE)  C_10_H_11_ClN_4_ |  | 222.68 | - | 0.62 | 189.6 |

# Octanol-water distribution coefficient (log K_ow_) and molar volume (V_m_) have been determined with the software ACD/ChemSketch

pK_a_ value for IMI obtained from Chamberlain et al. (1996)

**Table S2** Quality parameters of selected secondary effluents

|  | **SEA** | **SEB** |
| --- | --- | --- |
| pH | 8.2 | 7.8 |
| Conductivity (μS/cm) | 850 | 615 |
| A_254_ (cm^-1^) | 0.101 | 0.169 |
| DOC (mg/L) | 4.3 | 9.4 |
| COD (mg/L O_2_) | 10 | 25 |
| Alkalinity (mg/L CaCO_3_) | 244 | 183 |
| Total nitrogen (mg/L N) | 2.72 | 5.6 |
| Total phosphorus (mg/L P) | 1.60 | 0.31 |

**Figure S1** Decay of Fe(VI) with reaction time in experiments of oxidation of THM by Fe(VI) in UP water at different pH. Experimental conditions: Initial concentration of THM = 1 μM; Initial concentration of Fe(VI) = 50 μM; T = 20 ºC

**Figure S2** Evolution of (A) THM, (B) IMI, (C) CLO, (D) THC and (E) ACE with reaction time during Fe(VI) treatment in UP water at different pH. Experimental conditions: Initial concentration of each neonicotinoid = 1 μM; [Fe(VI)]_0_ = 100 μM; T = 20 ºC

**Figure S3** Evolution of THC with reaction time during Fe(VI) treatment in UP water, secondary effluents and in the presence of HA. Experimental conditions: Initial concentration of each neonicotinoid = 1 μM; [Fe(VI)]_0_ = 100 μM; T = 20 ºC; pH ≈ 8

**Figure S4** Evolution of (A) THM, (B) IMI, (C) CLO, (D) THC and (E) ACE with reaction time during Fe(VI)/PMS treatment in UP water with different concentrations of reagents. Experimental conditions: Initial concentration of each neonicotinoid = 1 μM; pH = 8; T = 20 ºC; initial Fe(VI) concentration settings used: 0, 50, 100 μM; initial PMS concentration settings used: 0, 100, 200, 400 μM

**Figure S5** Evolution of (A) THM, (B) IMI, (C) CLO, (D) THC and (E) ACE with reaction time during Fe(VI)/PMS treatment in UP water at different pH. Experimental conditions: Initial concentration of each neonicotinoid = 1 μM; [Fe(VI)]_0_ = 50 μM; [PMS]_0_ = 200 μM; T = 20 ºC

**Figure S6.** Evolution of (A) THM, (B) IMI, (C) CLO, (D) THC and (E) ACE with reaction time during Fe(VI)/PMS treatment in UP water in the presence of the radical scavengers TBA and MeOH. Experimental conditions: Initial concentration of each neonicotinoid = 1 μM; [Fe(VI)]_0_ = 50 μM; [PMS]_0_ = 200 μM; T = 20 ºC

**Figure S7** Degradation of neonicotinoids by the Fe(VI)/sulfite system in UP water at different pH: instant removal percentages of each neonicotinoid. Experimental conditions: Initial concentration of each neonicotinoid = 1 μM; [Fe(VI)]_0_ = 50 μM; [sulfite]_0_ = 200 μM; T = 20 ºC

**Figure S8** Degradation of neonicotinoids by the Fe(VI)/sulfite system in UP water in the presence of the radical scavengers TBA and MeOH and under N_2_ atmosphere: instant removal percentages of each neonicotinoid. Experimental conditions: Initial concentration of each neonicotinoid = 1 μM; [Fe(VI)]_0_ = 50 μM; [sulfite]_0_ = 200 μM; pH = 8; T = 20 ºC

**References**

Acero JL, Real FJ, Benitez FJ, Matamoros E (2019) Degradation of neonicotinoids by UV irradiation: Kinetics and effect of real water constituents. Sep Purif Technol 211:218–226. https://doi.org/10.1016/j.seppur.2018.09.076

Chamberlain K, Evans AA, Bromilow RH (1996) 1-Octanol/water partition coefficient (K_OW_) and pK_a_ for ionisable pesticides measured by a pH-metric method. Pestic Sci 47:265–271. https://doi.org/10.1002/(SICI)1096-9063(199607)47:3<265::AID-PS416>3.0.CO;2-F

Real FJ, Acero JL, Benitez FJ, Matamoros E (2022) Elimination of neonicotinoids by ozone-based advanced oxidation processes: Kinetics and performance in real water matrices. Sep Purif Technol 301:121975. https://doi.org/10.1016/j.seppur.2022.121975
